# The effect of changing from campus-based to digital teaching on student attendance: A case study of Norwegian business students

**DOI:** 10.1016/j.heliyon.2022.e11307

**Published:** 2022-11-02

**Authors:** Erik Haugom

**Affiliations:** Inland Norway University of Applied Sciences, Lillehammer Campus, Norway

**Keywords:** Covid-19, Digital teaching, Online learning, Attendance rate, Higher education

## Abstract

Over the last two years, the Covid-19 pandemic has forced colleges, university colleges, and universities around the world to switch from campus-based to web-based teaching. The current study examines the effect on students' attendance rates from this sudden change in the learning environment. Data from four completions of a single course given to third-year bachelor students in the business administration program at a mid-sized university college in Norway were used to empirically examine how the level- and trend of the attendance rate were affected during the lockdown period. By means of regression analysis, the results show that digital lectures had a significantly higher attendance rate compared with traditional campus-based lectures. No significant difference in the trends of the attendance rate over the semester was found between the two lecture forms.

## 1 Introduction

Attendance is important to student success measured by academic performance (Arulampalam et al., 2012; Credé et al., 2010; Dobkin et al., 2010; Kirby and McElroy, 2003; Stanca, 2006) and retention (see e.g., Bowen et al., 2005). According to Tran and Gershenson (2021), there is also a growing consensus that student attendance is an important input in the education production function, and as such the economics of education. Higher attendance rates thus have both short- and long-term benefits for individual students, educational institutions, and society in general. Hence, attendance rates are an ongoing concern among policymakers, faculty, and management within higher education.

The Covid-19 pandemic forced most higher education institutions (HEIs) around the world to move from campus-based to online teaching more or less ‘over the night’ (Vital-López et al., 2022; Zamora-Antuñano et al., 2022). The digital transformation was, in many cases, implemented with limited insights into the potential consequences related to the students' attendance rates. Zamora-Antuñano et al. (2021) for example reported that exactly student attendance was one of the main concerns teachers identified during the Covid-19 crisis. How would the move from physical to digital teaching affect the number of students showing up for class?

The question above has yet not been addressed in the literature. It is an important question though because of the link between attendance, academic performance, retention, and the economics of education, and because the answer can influence policymakers' decisions in similar situations in the future.

Student attendance has been studied from many angles in previous research. In a recent review of extant research on the topic, Moores et al. (2019) group the determinants of attendance and absenteeism into five broad categories: 1) teaching issues (e.g., Tran and Gershenson, 2021), 2) effects of university expectations and policy (e.g., Dickson and Stephens, 2016), 3) scheduling issues (e.g., Devadoss and Foltz, 1996; Kelly, 2012), 4) provisions of online material (e.g., Grabe, 2005), and 5) individual factors outside HEI's control (e.g., Oldfield et al., 2018).

The first category, teaching issues, is related to teaching quality and style and is, according to Moores et al. (2019), difficult to quantify objectively. In the present study, the same lecturer provided all the lectures over the entire sample period. Hence, there should be no systematic variation in variables reflecting teaching issues.

The category closest related to the current study is ‘provisions of online material’. Research in this category has typically focused on what effect the provision of lecture-slides and recordings online has on attendance (e.g., Babb and Ross, 2009; Copley, 2007; Larkin, 2010; Traphagan et al., 2010). Though the evidence is somewhat mixed, many previous studies indicate that the provision of online material in general seems to decrease attendance.

The provision of online material to support student learning is something different than switching completely from campus-based teaching to digital teaching though. When offering digital material as support, the lecturer is still physically present in the sessions taking place on campus. Mattick et al. (2007) compared attendance rates at a live lecture location where the lecturer was physically present with attendance at a remote site where the same lecture was delivered via video link but found no significant differences. To the best of my knowledge, no prior studies have examined the effect of switching from a campus-based to a fully digital version directly.

The main objective of the current study is therefore to fill this gap by offering some initial empirical evidence on how the switch from campus-based to digital teaching affected attendance in higher education. To this end, I use attendance data collected over four complete run-throughs of a course taught to third-year bachelor business students at a mid-sized university college in Norway. One out of the four run-throughs was fully taught on Zoom (a digital platform for teaching and meetings), while the other three were given on campus with traditional campus-based teaching methods. The results show that (1) the general attendance level was significantly higher on Zoom vs on campus, and (2) the negative trend over the semester was stronger in the on-campus case vs on Zoom, though the difference in the trends was not statistically significant.

The rest of the paper is organized as follows. The next section describes the data and offers some summary statistics of the study variables. Then the empirical results are presented followed by a section presenting the conclusion, implications, and future research.

## 2 Data description

The data consist of attendance numbers recorded by the lecturer from 2018 to 2021 (four complete run-throughs) of a single course given to third-year bachelor business students at a mid-sized university college in Norway. In the fall semester of 2020, the course was fully digital and taught via the Zoom cloud meetings platform due to public health policies in Norway. Additionally, because of the same policies and university guidelines, three lectures at the beginning of the 2021 run-through were taught via Zoom making the total number of digital lectures 17. The course syllabus was identical in all four run-throughs, apart from four workshop lectures given in the on-campus version each semester, which was not offered in the online case. I control for this difference in the empirical analysis by including a dummy variable for the workshop lectures. The course has been held on the same weekdays (Wednesday and Thursday) and time slots (8.15–10.00) over the entire sample period. Hence, scheduling effects related to day-of-the-week and time-of-the day (see e.g., Kelly, 2012) should not be an issue in the current study. Many previous studies have shown a negative trend in attendance over the term (Burd and Hodgson, 2006; Colby, 2005; Davis et al., 2012; Mattick et al., 2007; Newman-Ford et al., 2008; van Blerkom, 1992). A variable that simply counts the number of days any given lecture took place within each term was therefore constructed. To test differences in the trend in the on-campus version vs. the digital version I used an interaction term between the digital dummy and the variable counting the days into the semester. Moores et al. (2019) point out that revision lectures at the end of the term are somewhat immune from the negative trend and a dummy variable to control for this potential effect was therefore constructed. The number of registered students to the course was counted when the second mandatory hand-in was due each year.

**Figure 1:**
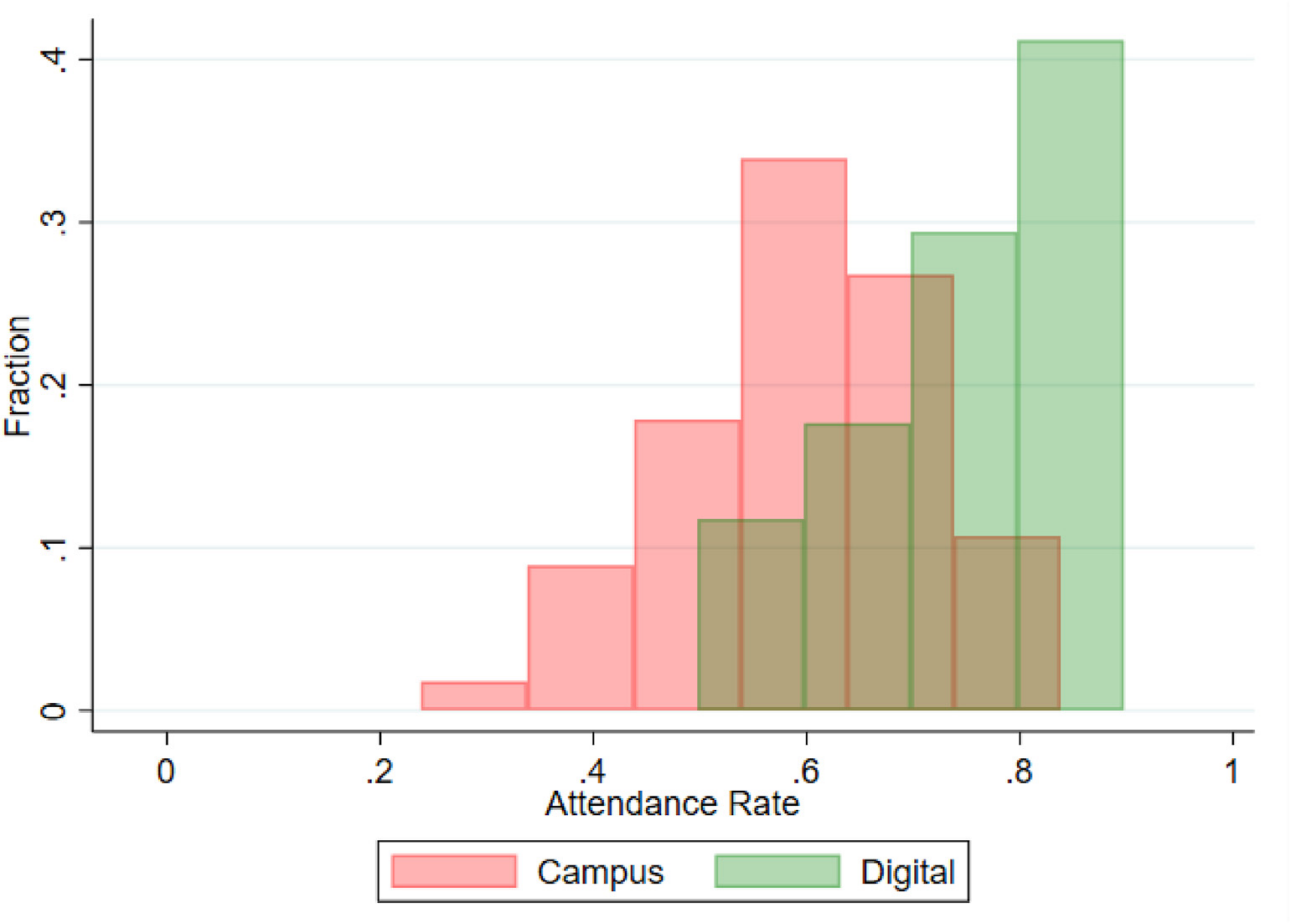
Histograms of attendance rates for campus-based- (red) and digital (green) lectures over the term.

**Figure 2:**
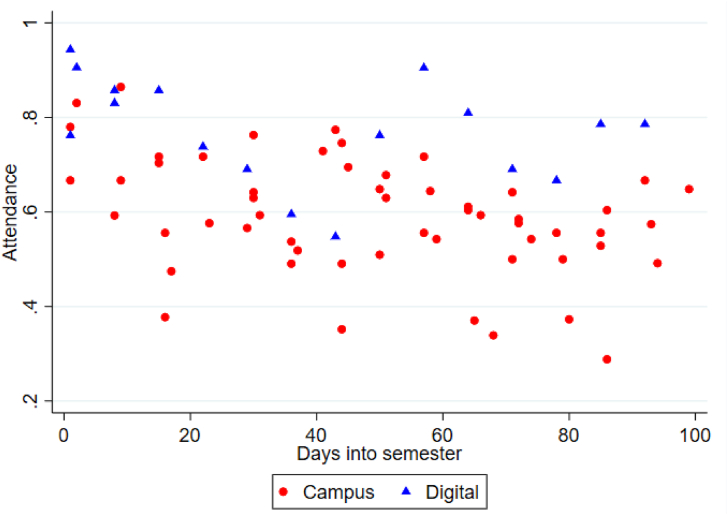
Attendance rate for campus-based- (red dots) and digital (blue triangles) lectures over the term.

Table 1, Figures 1 and 2 summarize the attendance rate over the entire sample period and highlights the difference between campus-based- and digital versions of the course. Table 1 shows that the average attendance rate was higher in the digital version compared with the campus-based version of the course. On campus (N = 56) the average attendance rate was 59.1% while the corresponding number on Zoom (N = 17) was 77.2%. Table 1 also shows that the variation around the mean is somewhat higher on campus with a wider minimum-maximum range and a higher standard deviation. This finding can be partly explained by the workshop lectures offered in the on-campus version, which is controlled for in the empirical modeling.

**Table 1:** Summary statistics of attendance rate.

	Attendance rate: Campus and digital				
	N	Mean	Max	Min	SD
Campus	56	0.591	0.864	0.288	0.123
Digital	17	0.772	0.943	0.548	0.110
Total	73	0.634	0.943	0.288	0.142

The summary statistics are further supported by histograms in Figure 1 to show the frequency distribution for the two lecture types. The figure clearly shows that campus-based lectures have a lower expected value and exhibit greater variation when compared to digital lectures. In fact, more than 40% of the digital lectures have an attendance rate of 80% or more in the sample.

Figure 2 illustrates attendance rates together with semester maturity measured in days from the first lecture of the course was given. The figure uses unique marker symbols for campus-based- (red circles) and digital (blue triangles) teaching to highlight potential differences in the data. The visual inspection of Figure 2 confirms the findings from Table 1 and Figure 1 that the general attendance level in the digital case is higher compared with the campus-based version. We also see indications of a somewhat stronger negative trend in the campus case, but this needs to be formally examined in an empirical model.

## 3 Empirical results

To examine how the attendance rate was affected by changing from campus-based to digital teaching I estimate three linear regression models of the following form^1^:

(1) $$AR=\alpha +\beta_{1}DIGITAL+\varepsilon$$

(2) $$AR=\alpha +\beta_{1}DIGITAL+\beta_{2}DAYS+\beta_{3}WS+\beta_{4}RL+\varepsilon$$

(3) $$AR=\alpha +\beta_{1}DIGITAL+\beta_{2}DAYS+\beta_{3}WS+\beta_{4}RL+\beta_{5}DIGITAL\;\times DAYS+\varepsilon$$

where AR is the attendance rate measured in percent of the total number of enrolled students, DIGITAL is a dummy variable taking the value one if the teaching was given on Zoom (zero otherwise), DAYS is the number of days from the first lecture of the course in the given semester, WS is a dummy variable for the workshop lectures, RL is a dummy variable for the revision lectures, and DIGITAL×DAYS is an interaction variable capturing whether the trend is different for the digital version of the course. Although the dependent variable is by definition limited in range (from zero to one), I report the standard linear specification because of easier interpretation of the results (see Angrist and Pischke (2009) for a justification of this choice). Logit specifications were also tested as a robustness check and the results are almost identical to the linear models.

The estimation results are reported in Table 2. Model 1 is based on Eq. (1) and only includes the digital lecture dummy variable and examines whether there is a difference in the general attendance rate between regular campus-based- and online Zoom-lectures. The results show a positive and statistically significant effect. The digital lectures have 18.1 percentage points higher attendance rates compared with campus-based lectures, on average. Including only the digital lecture dummy variable explains almost 30% of the variation in the attendance rate ($R^{2}=29.5\%$). Model 2 is based on Eq. (2) and controls for a potential trend in the attendance rate over the term and workshop and revision lectures. The main study variable (digital lecture) is still positive and highly significant when including the control variables, though the effect is somewhat reduced. The average attendance rate for the digital lectures is 13.8 percentage points higher than campus-based lectures when including the control variables. The results from Model 2 also show a significant negative trend over the term. For each day from course start the attendance rate drops by approximately 0.17 percentage points. This means that a month into the term the attendance rate was approximately 5 percentage points lower, on average. This finding is in line with previous results in the literature (e.g., van Blerkom, 1992). Workshop lectures have 12.9 percentage points lower attendance rate compared with regular lectures, on average. The effect is significant. Revision lectures have almost 6 percentage points higher attendance on average, but this effect is not statistically significant.

**Table 2:** OLS estimates of attendance rate by digital lecture and control variables.

	(1)	(2)	(3)
VARIABLES	Model 1	Model 2	Model 3
Digital lecture (1 = yes; 0 = no)	0.1810∗∗∗	0.1378∗∗∗	0.1187∗∗∗
	(0.0309)	(0.0289)	(0.0440)
Days into semester		-0.0017∗∗∗	-0.0019∗∗∗
		(0.0005)	(0.0005)
Workshop lecture (1 = yes; 0 = no)		-0.1292∗∗∗	-0.1288∗∗∗
		(0.0423)	(0.0430)
Revision lecture (1 = yes; 0 = no)		0.0595	0.0592
		(0.0428)	(0.0416)
Digital × Days into semester			0.0005
			(0.0008)
Constant	0.5914∗∗∗	0.6983∗∗∗	0.7044∗∗∗
	(0.0165)	(0.0246)	(0.0287)
Observations	73	73	73
R-squared	0.295	0.514	0.515
Mean VIF	1.00	1.15	2.01
Jarque-Bera normality test	1.044	0.324	0.332

Heteroscedasticity and autocorrelation robust standard errors in parentheses (Andrews, 1991). The Jarque-Bera test follows the chi-square distribution with two degrees of freedom. The critical value at the 5% significance level is 5.99.

∗∗∗p < 0.01, ∗∗p < 0.05, ∗p < 0.1

Model 3 is based on Eq. (3) and includes an interaction term between digital teaching and the number of days into the semester to examine whether there also is a difference in the trend in the digital vs the campus-based version of the course. The estimated coefficient is positive, but not statistically significant. Hence, the empirical results show some indications of a weaker negative trend over the term in the case of online Zoom lectures, but the difference in the two trend estimates is not statistically significant. The other effects reported in Model 2 still hold and change little when including the interaction term. The variance inflation factor (VIF) and the Jarque-Bera test for normality indicate that multicollinearity and non-normality are not an issue in any of the model specifications.

## 4 Conclusion, implications, and future research

The current study has examined the effect of moving from campus-based to digital teaching on attendance rates. The data stem from four complete run-throughs of the same course, with the same lecturer, held on the same weekdays, and timeslots every year. The 2020 run-through and three lectures in 2021 were provided online via the Zoom platform. The data are thus similar to those obtained with an experiment. The only things varying systematically is whether the course was offered digitally (Zoom) or on-campus and the presence of workshop lectures.

The empirical results show that the digital lectures had a significantly higher attendance rate compared with the traditional campus-based lectures. There is also an indication of a steeper decline in the attendance rate over the term in the on-campus case compared with the lectures offered fully online, but the difference in the two trends is not statistically significant. Digital lectures are more flexible than campus-based lectures as students can follow them from anywhere in the world. Such digital lectures also require less effort to attend compared to the campus-based alternative. The provision of digital material has previously been documented to have a negative effect on physical attendance. Grabe (2005) for example found that 50% of frequent online note users indicated they used these as a replacement for class attendance between one and five times over the duration of the course. As many as 29% of the students reported they used the online notes as a replacement for attendance six times or more. Traphagan et al. (2010) reported that 31% of students having access to webcasts “often” or “always” used these instead of attending class. A possible explanation for the higher attendance rate, and weaker negative trend (though not significant), in the digital case, is therefore convenience and easier access. This reasoning is partly also supported by the findings of Mattick et al. (2007) who did not find any significant differences in attendance for lectures where the lecturer was physically present and the same lectures delivered via video link at a remote site. In that case, the students in both groups had to meet at a physical location to attend the lecture and following the lecture digitally is no longer more convenient. Hence, there is a distinction between online material as additional (voluntary) learning resources and digital lectures as the only available alternative. The results of the present study show that attendance can increase in the latter case.

Policymakers, faculty, and management within higher education can use the findings reported here as decision support in the future. The study is not without limitations though, and a plethora of other potential consequences of moving from campus-based to fully digital teaching must be considered in that case of course. Examples include: (1) how actively students participate in the lectures, (2) how students meet the intended learning outcomes, and (3) the long-term psychological effects of not being able to physically interact with fellow students and faculty members. The results of the present study are also limited to one course on a given business program. The findings can therefore not be generalized to other courses or study programs. Future research should examine whether the effects hold across multiple courses and study programs and how the switch to digital teaching affects academic performance, retention, and the economics of education in general.

## Declarations

### Author contribution statement

Erik Haugom: Conceived and designed the experiments; Performed the experiments; Analyzed and interpreted the data; Contributed reagents, materials, analysis tools or data; Wrote the paper.

### Funding statement

This research did not receive any specific grant from funding agencies in the public, commercial, or not-for-profit sectors.

### Data availability statement

Data will be made available on request.

### Declaration of interest’s statement

The authors declare no conflict of interest.

### Additional information

No additional information is available for this paper.

## Footnotes

1 The attendance rate is bounded between zero and one, but the linear method works very well also in most such cases and is actually preferred for causal inference (see Angrist and Pischke, 2009).
